# Innovative Mobile App (CPD By the Minute) for Continuing Professional Development in Medicine: Multimethods Study

**DOI:** 10.2196/69443

**Published:** 2025-07-23

**Authors:** Peter Slinger, Maram Omar, Sarah Younus, Rebecca Charow, Michael Baxter, Craig Campbell, Meredith Giuliani, Jesse Goldmacher, Tharshini Jeyakumar, Inaara Karsan, Janet Papadakos, Tina Papadakos, Alexandra Jane Rotstein, May-Sann Yee, Asad Siddiqui, Marcos Silva Restrepo, Melody Zhang, David Wiljer

**Affiliations:** 1 University Health Network Toronto, ON Canada; 2 Temerty School of Medicine University of Toronto Toronto, ON Canada; 3 Ontario Health Toronto, ON Canada; 4 St Joseph's Health Centre Toronto, ON Canada; 5 Faculty of Medicine University of Ottawa Ottawa, ON Canada; 6 Princess Margaret Cancer Centre University Health Network Toronto, ON Canada; 7 The Wilson Centre Toronto, ON Canada; 8 Foothills Medical Centre Calgary, AB Canada; 9 University of Calgary Calgary Canada; 10 Southlake Health Newmarket, ON Canada; 11 The Hospital for Sick Children Toronto, ON Canada; 12 Sunnybrook Health Sciences Centre Toronto, ON Canada; 13 Mastercard Foundation Toronto, ON Canada; 14 Institute of Health Policy, Management & Evaluation University of Toronto Toronto, ON Canada

**Keywords:** continuing professional development, mobile app, question-based learning, lifelong learning, self-assessment, artificial intelligence

## Abstract

**Background:**

Many national medical governing bodies encourage physicians to engage in continuing professional development (CPD) activities to cultivate their knowledge and skills to ensure their clinical practice reflects the current standards and evidence base. However, physicians often encounter various barriers that hinder their participation in CPD programs, such as time constraints, a lack of centralized coordination, and limited opportunities for self-assessment. The literature has highlighted the strength of using question-based learning interventions to augment physician learning and further enable change in practice. CPD By the Minute (CPD-Min) is a smartphone-enabled web-based app that was developed to address self-assessment gaps and barriers to engagement in CPD activities.

**Objective:**

This study aimed to assess the app using four objectives: (1) engagement and use of the app throughout the study, (2) effectiveness of this tool as a CPD activity, (3) relevance of the disseminated information to physicians’ practice, and (4) acceptability to physicians of this novel tool as an educational initiative.

**Methods:**

The CPD-Min app disseminated 2 multiple-choice questions (1-min each) each week with feedback and references. Participants included licensed staff physicians, fellows, and residents across Canada. A concurrent multimethods study was conducted, consisting of preintervention and postintervention surveys, semistructured interviews, and app analytics. Guided by the Reach, Effectiveness, Adoption, Implementation, and Maintenance framework, the qualitative data were analyzed deductively and inductively.

**Results:**

Of the 105 Canadian anesthesiologists participating in the study, 89 (84.8%) were staff physicians, 12 (11.4%) were fellows, and 4 (3.8%) were residents. Participants completed 110 questions each over the course of 52 weeks, with an average completion rate of 75% (SD 33%). In total, 40.9% (43/105) of participants answered >90% of the questions, including 15.2% (16/105) who completed all questions. Moreover, 69% (52/75) of participants reported the app to be an effective and valuable resource for their practice and to enhance continuous learning. Most participants (63/75, 84%) who completed the postsurveys reported that they would likely continue using the app as a CPD tool. These findings were further supported by the interview data. Three key themes were identified: the practical design of the novel educational app facilitates its adoption by clinicians, the app was perceived as a useful knowledge tool for continuous learning, and the app’s low-stakes testing environment cultivated independent learning attitudes.

**Conclusions:**

The findings suggest the potential of the app to improve longitudinal assessments that promote lifelong learning among clinicians. The positive feedback and increased acceptance of the app supports it as an innovative tool for knowledge retention and CPD. Future research efforts should prioritize evaluating the app’s long-term sustainability and its impact on physicians’ practice, as well as exploring alternative approaches (such as artificial intelligence–based tools) for generating questions.

## Introduction

### Background

According to the Accreditation Council for Continuing Medical Education, continuing medical education (CME) supports physicians and health care teams in their lifelong learning and engagement in self-directed learning and quality improvement, leading to better care for patients and communities [1]. Continuing professional development (CPD) learning activities generally consist of a selection of educational and developmental activities, including group learning, self-directed learning, and self-assessment activities [1,2]. Such CPD activities for specialist physicians in Canada are overseen by the Royal College of Physicians and Surgeons (RCPSC) using the Maintenance of Certification (MOC) program [3]. Traditional CPD activities have been shown to have a limited impact on physicians’ performance and patient outcomes [3]. Furthermore, experts suggest that systems-integrated CME activities that use a longitudinal multimodal approach allow for organizational improvements, as well as positive change in practice and patient health outcomes [4,5]. However, implementing longitudinal CME is not without challenges, including limited availability of objective practice data, inaccurate physician self-assessment, and ineffective CPD activities [5-7].

Longitudinal assessments have successfully been used in medical education where multiple-choice and short-answer questions are delivered at spaced intervals on a computer or mobile device [5,8]. More recently, this type of longitudinal assessment has been used for CPD of physicians [5,8]. One approach that has been shown to be effective in supporting clinicians’ CPD is question-based learning interventions, such as test-enhanced learning (TEL) [9]. TEL has been shown to promote knowledge retention and information retrieval from memory [9]. As part of the learning sciences, spaced repetition and intentional recall are effective techniques for enhancing knowledge retention [10-13]. The process of recalling information for testing purposes further consolidates the material in the long-term memory and helps strengthen the memory trace when it is repeatedly tested [13]. In addition to repeated testing, feedback after retrieval attempt has been shown to increase the effects of TEL, as it reinforces the correct material in the long-term memory and retention [14]. This can promote reflective learning, as health care professionals can identify their strengths, weaknesses, and knowledge gaps that require further learning. Several studies have found that incorporating TEL and spaced repetition in CME programs enhances knowledge retention among clinicians [15-17].

The American Board of Anesthesiology (ANES) has developed a program of web-based multiple-choice questions (MCQs) that have been used for recertification in ANES, replacing their recertification examination [18]. This type of recertification allows ANES health care professionals to stay up-to-date on the latest developments and best practices in their field, improving their performance and patient outcomes. Furthermore, the use of this web-based MCQs CME provides an opportunity to be integrated into organizations and organizational workflows, as an effective strategy for promoting ongoing learning and development among health care professionals [1]. However, the question of how this approach could be used beyond the MOC remains largely unanswered. What is the role of digital approaches such as this one in the promotion of self-assessment, reflective learning, personalized learning, lifelong learning, and practice improvement?

Digital tools have increasingly been adapted to support pedagogies for CPD and have the potential to address limitations of self-assessment identified in existing CPD programs for specialist physicians in Canada [19,20]. Self-assessment CPD tools effectively promote lifelong learning through spaced longitudinal question-based assessments and reflective learning [4]. A team of clinicians and researchers at the University Health Network (UHN) developed a web- and mobile-based app, called CPD By the Minute (CPD-Min), that disseminates weekly self-assessments to specialist clinicians in Canada. A prototype of this progressive web-based app was initially developed by the team to assess the feasibility and utility of this concept in a Canadian setting. The prototype was first tested by 17 members of the University of Toronto’s (UofT) Department of Anesthesiology to demonstrate the feasibility of the app and to evaluate user experience, educational experience, and perceived efficacy [21]. The mobile app format was positively received, with 88% of users likely to continue using the app and 76% of users citing the app as an effective learning tool [21]. The tool was recognized for its ease of use, accessibility, and minimal time commitment. Users suggested the addition of a feedback feature to allow them to compare their performance to a peer group [21].

### This Paper

On the basis of the feedback from the feasibility study, the CPD-Min pilot study was launched to further explore the efficacy of the app among ANES clinicians. This paper assesses the CDP-Min app using the four objectives related to the deployment of CDP-Min in the ANES context: (1) the effectiveness of this tool as a CPD activity; (2) ANES residents’, fellows’, and staffs’ acceptability of this novel tool as an educational initiative; (3) relevance of the disseminated information to physicians’ practice; and (4) engagement and use of the app throughout the study.

## Methods

### Study Design

The CPD-Min pilot study was a prospective, concurrent multimethods study, with assessment components implemented throughout the study. This multimethod evaluation of the app was guided by the Reach, Effectiveness, Adoption, Implementation, and Maintenance (RE-AIM) framework in the development of the survey and interview questions [22]. The RE-AIM framework was used to assess the design and development of the app as well as the efficacy of the intervention.

### Ethical Considerations

This study was approved by the UHN Research Ethics Board (20-5883), the UofT Research Ethics Board (00040879), and the UofT CPD committee for CME credits. Participants in the study were required to review and sign the consent form sent via email by the study coordinator. The consenting process specified that participation entails agreeing to the terms of use and engaging with the CPD-Min application for the entire study duration, completing preintervention and postintervention surveys, and participating in an optional follow-up interview. The consent form outlined the voluntary nature of participating in this study, the benefits and risks, and data handling and storage. Participants were able to withdraw from the study at any time without penalty. The survey and interview data gathered were stored on confidential databases on secured UHN servers or encrypted and password-protected UHN devices. Interview transcripts were deidentified before analysis. After the transcripts have been verified for accuracy and data coding is complete, the audio recordings were destroyed. Data from the surveys were entered into a database on the UHN network shared drive, where only study team members have access. Only aggregated and thematically analyzed data were shared with study collaborators.

### Education Intervention

The CPD-Min app was developed at UHN by a multidisciplinary team of clinicians, researchers, and software developers. Before the launch of the study, members of the research team (ie, experts and associate deans with relevant academic and professional backgrounds in the field of CPD) engaged in usability testing to assess and approve the app’s design.

#### Description of the CPD-Min App

The CPD-Min app is a smartphone-enabled web-based app that administered 2 questions (1 min each) each week over a 52-week period. Following participants’ response to the question, the app provided detailed feedback, including the correct answer, critiques of the wrong answer, key points, and references. This provided the participants with an opportunity to identify gaps in their knowledge and further explore these topics using the resources provided. In addition to answering the questions, participants were asked to rate each question based on their confidence (ie, very confident, somewhat confident, or not at all confident) and its relevance (ie, very relevant, somewhat relevant, or not relevant) to their practices. Moreover, an aggregated summary of all participants’ responses and reflection ratings of each question were available for them to review after each week on the app’s dashboard. This feature allowed participants to compare their performance to their peers and reflect on their performance accordingly. Finally, 2 knowledge assessments were conducted, at the midpoint (T1) and the end point (T2) of the study, where 5 questions were recycled back to participants if answered incorrectly or omitted.

#### Question Development

**Figure 1:**
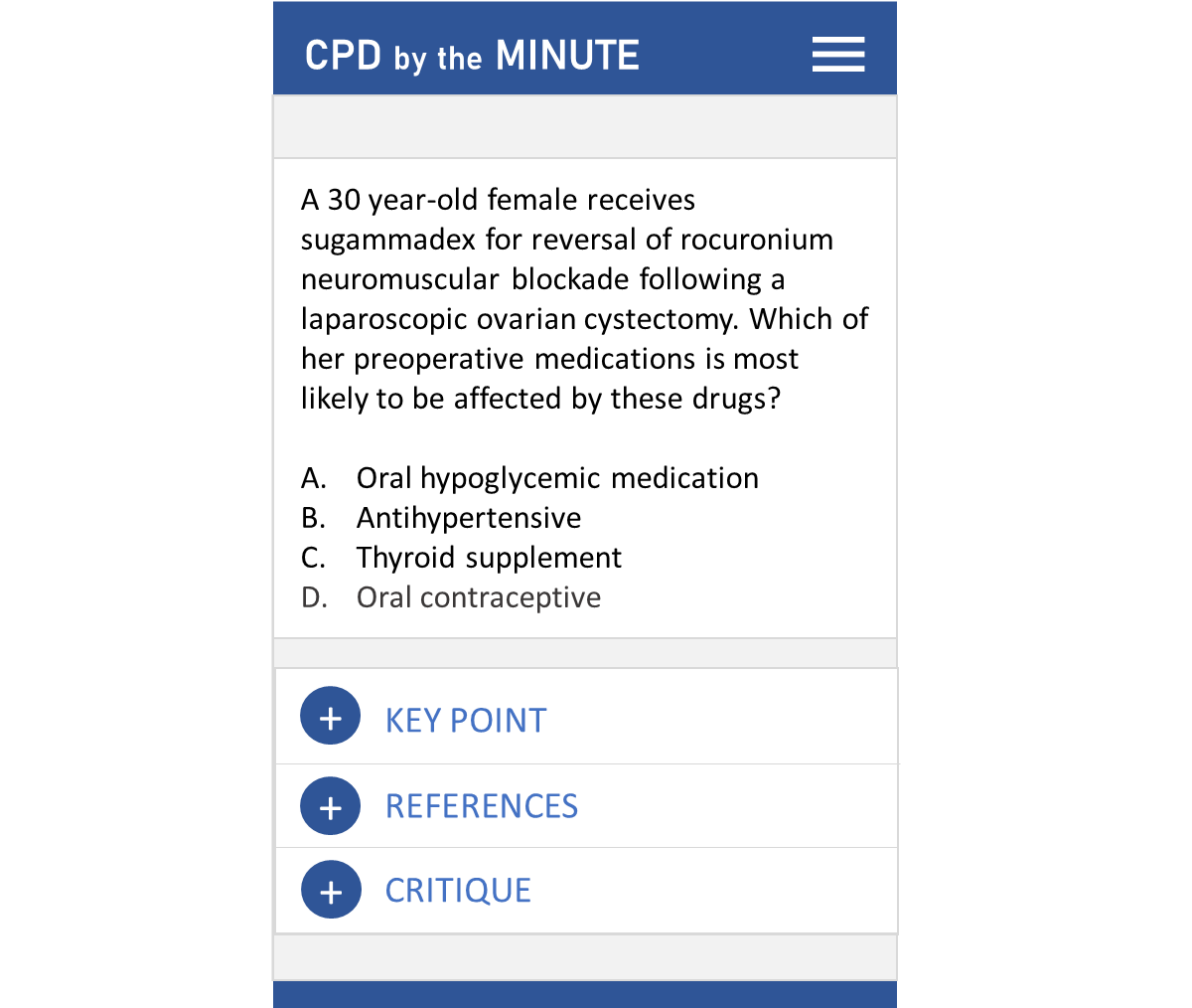
A CPD By the Minute mock-up of a clinical pharmacology question for anesthesiology.

A total of 7 residents and staff from the UHN and UofT ANES departments were recruited to assist with question development; research associates provided guidance to ensure the consistency and quality of questions. A peer-review process involving 2 content experts was then conducted to validate, refine, and provide feedback on the content, quality, and relevance of the questions to CPD. Each question was multiple choice and contained a question stem, 4 to 5 answer choices, a key point, a critique of incorrect answers, and references (Figure 1). Questions developed were used to assess health care professionals’ knowledge of important practice standards and guidelines and to stimulate problem-solving in 23 general and specific subspecialty topics, such as critical care and resuscitation, cardiology and cardiovascular surgery, neurology and neurosurgery, and general complications. The MCQs developed were a mixture of clinically related case-based questions as well as calculation and statistical questions. Approximately 100 ANES-based questions were developed and reviewed. The number of questions developed was based on the size of the anesthesiology specialty in Canada. A Microsoft OneNote database was created to allow for collaboration and monitoring of questions developed in real time. The questions were developed iteratively over a 6-month period to disseminate new practice information as the emerging needs of clinical practice continuously evolve.

#### App Development and Usability Testing

Usability testing was conducted to assess and approve the design of the CPD-Min app as well as to identify opportunities to improve the user experience before the release of the final version of the app and the launch of the pilot study, thereby optimizing the app for better user engagement. A total of 7 experts and associate deans with relevant academic and professional backgrounds in the field of CPD were recruited for usability testing (including a pre- and postsurvey) via purposive sampling. Each testing session took place, via Microsoft Teams, due to COVID-19 public health restrictions. Furthermore, the Post Study System Usability Questionnaire, including 3 subscales—system usefulness, information quality, and interface quality—was administered after the usability test to evaluate the usability of the app and their perceived satisfaction. Data from the sessions were evaluated using the 10 Usability Heuristic Principles and Severity Scale by Nielsen [23].

### Recruitment

Recruitment materials (email invitations with consent forms, digital posters, and a recruitment video) developed by the research team were sent out to anesthesiologists through the UofT’s departments of ANES and specialty society organizations (Association of Canadian University Departments of Anesthesia, Continuing Education and Professional Development) across Canada, where a snowball sampling approach was used for recruitment.

Before conducting the study, participating ANES physician staff, fellows, and residents gave their written informed consent via electronically signed documents. The study was conducted in 2 sequential cohorts over a 52-week period, initiated in October (cohort 1) and November 2021 (cohort 2).

### Data Collection

**Figure 2:**
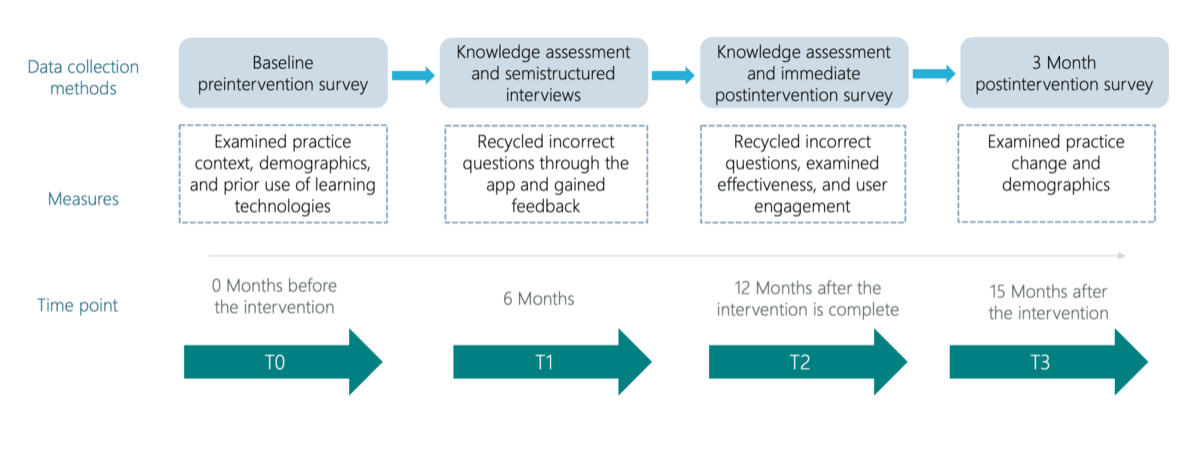
Data collection tools and measures.

Data collection occurred throughout 4 distinct time points of the study (T0, T1, T2, and T3) and included electronic preintervention and postintervention surveys, knowledge assessments, and semistructured interviews with participants who had consented (Figure 2). The 3 surveys (preintervention, postintervention and 3 months after the intervention) were administered via REDCap (Research Electronic Data Capture; Vanderbilt University). The preintervention survey was administered before participants’ engagement with the app (T0), and it included questions on practice context and demographics and prior use of learning technologies.

At the midpoint of the study (T1), semistructured interviews were conducted and recorded on Microsoft Teams with participants from ANES who provided consent. They were on average 17 (SD 0.29) minutes in duration, with questions on acceptability, relevance, expansion, and potential impact of CPD-Min. At weeks 26 and 52, the app recycled a maximum of 5 questions back to participants based on previous user responses using a random selection process. If ≥1 question was answered incorrectly before the first knowledge testing time point (T1), a maximum of 5 questions were randomly selected from a list of incorrectly answered questions for a second attempt. Recycled questions that were either omitted or incorrectly answered at T1 were carried forward as part of the pool or list of eligible questions to be revisited once more during the final knowledge testing week (T2). For the users who exhibited perfect scores at either T1 or T2, there was no knowledge testing. In addition, app analytics (eg, number of questions answered, correct questions, and time spent answering questions) were collected throughout the 52-week study period.

The postsurvey was sent to participants at the end of the pilot program (T2), and it included questions on the effectiveness and appeal of the app, influence of peer comparison, user engagement behavior, quality assessment, app design, and the System Usability Scale (SUS; Multimedia Appendix 1). The questions were designed by the research team based on peer-reviewed literature, and a subset of them was pilot-tested during the feasibility study. The SUS is a widely used 10-item questionnaire designed to assess the usability and effectiveness of a digital tool [24]. For example, participants rated various aspects of the app, such as the ease of use, functionality integration, the need for technical support, and overall complexity, on a scale from 1 to 5, ranging from “strongly disagree” to “strongly agree.” A survey was sent to participants 3 months after the intervention (T3) with questions recapturing demographics and others exploring practice change or improvement.

### Data Analysis

#### Quantitative Analyses

All quantitative data were cleaned and analyzed using SPSS (IBM Corp). Preintervention and postintervention survey data were analyzed for descriptive statistics and correlational findings. With regard to the knowledge assessments, descriptive results were used to describe the extent of learning due to question recall. This included the number of correct or incorrect responses, the number of responses that timed out or were not started, question relevance, response times, and perceived confidence in answering questions. A series of paired 2-tailed *t* tests were conducted to compare knowledge assessments’ mean scores and evaluate change in question responses at different time points. In addition, Spearman rank correlation tests were conducted to assess the relationship between the proportion of participants who answered correctly or incorrectly and degrees of confidence and relevance. A *P* value of <.05 was used to determine significance throughout.

#### Qualitative Analysis

For the semistructured interviews, data were transcribed by a professional transcription service and cleaned for clarity and to remove any identifying information. Data were deductively and inductively analyzed using NVivo (Lumivero) following the thematic analysis process by Braun and Clarke [25]. Participant interviews were first deductively analyzed using the RE-AIM framework to code the data. Following the deductive analysis, the step-by-step process developed by Braun and Clarke [25] was used to inductively generate themes from the coded data by searching the data to capture participants’ thoughts and perceptions of the app and reviewing the preliminary themes before defining and describing them.

Finally, in addition to the RE-AIM framework being used to frame the findings from this pilot study, the continuing education outcome framework by Moore et al [26] was used to evaluate the learner’s experience and the app as a CPD activity (Table 1).

**Table 1:** CPD By the Minute (CPD-Min) outcome measures using the Reach, Effectiveness, Adoption, Implementation, and Maintenance (RE-AIM) framework.

RE-AIM framework components	Pilot study outcome measures
Reach	Number of American Board of Anesthesiology participants taking part in the CPD-Min program study Demographics of participants
Adoption	Participant completion rates were tracked throughout the 1-year pilot study
Effectiveness	Satisfaction using the CPD-Min app Mean scores from questions attempted throughout the program Mean scores from knowledge assessments
Implementation	Quality assessment of the content and delivery modes for the program
Maintenance	Prospective measure of self-reported continued use of the app Self-reported recommendation of the app

## Results

### Study Demographics: Reach

A total of 105 ANES participants were recruited for both cohorts to participate in the CPD-Min app study. Out of the 105 ANES participants in the study, there were varying response rates for the surveys administered preintervention, postintervention, and 3 months after the intervention. The response rate for the presurvey was 8.6% (9/105), while the response rate for the postsurvey was 71.4% (75/105). Due to the low response rate in the presurvey, a 3-month postsurvey (41/105, 39% response rate) was administered to gather information on demographics and practice change.

Of the 105 participants, there were 89 (84.8%) staff, 12 (11.4%) fellows, and 4 (3.8%) residents who took part in the study. Of the 89 staff, 40 (45%) reported on their years of practice with a variety of practice experience, but most reported <5 practice years (11/41, 27%) or 20 to <30 practice years (8/41, 20%). Most participants were in the age categories of 36 to 45 years (14/41, 34%) and 46 to 55 years (12/41, 28%), with most of them identifying as a cisgender man (33/41, 81%). Table 2 shows more details on participants’ demographics.

A large proportion of participants recruited were practicing in the Greater Toronto Area (eg, Mount Sinai, Humber River Hospital, Sunnybrook Health Sciences Centre, and UHN). Others were from hospitals and universities in Quebec, Manitoba, and British Columbia. Most participants (29/41, 71%) from the survey indicated that they practiced at an academic health sciences center or a teaching hospital, with varying participation in CPD activities, such as attending departmental rounds (39/41, 95%), meeting informally with colleagues (35/41, 85%), attending and presenting in conferences and courses (34/41, 83%), and many more (refer to Table 2 for the full list).

**Table 2:** Demographics and continuing professional development (CPD) activities of the CPD By the Minute app participants from the 3-month postsurvey (N=41).

Characteristics			Participants, n (%)
**Staff (years of experience)**			40 (98)
	<5	11 (27)	
	5 to <10	6 (15)	
	10 to <15	5 (12)	
	15 to <20	7 (17)	
	20 to <30	8 (20)	
	>30	3 (7)	
Fellow (year 3)			1 (2)
**Practice setting**			
	Academic health science center or teaching hospital practice	29 (71)	
	Community-based hospital practice	12 (29)	
**Age (years)**			
	26-35	6 (15)	
	36-45	14 (34)	
	46-55	12 (29)	
	56-65	7 (17)	
	>65	2 (5)	
**Gender**			
	Cisgender Man	33 (80)	
	Cisgender Woman	7 (17)	
	Transgender Person	1 (2)	
**Annually practiced CPD activities**			
	Attending formal case conferences to discuss patient care	29 (71)	
	Performing chart audits or reviewing performance data	14 (34)	
	Meeting informally with colleagues	35 (85)	
	Attending journal clubs	27 (66)	
	Attending departmental rounds	39 (95)	
	Attending and presenting in conferences and courses	34 (83)	
	Engaging in online activities or courses	33 (80)	

### Research Objective 1: Engagement and Use of the App Throughout the Study—Adoption

The *adoption* of the app was measured by looking at participants’ completion rate and use of the app throughout the 52-week pilot study (first-level of the framework by Moore et al [26] - participation). The app was assessed based on its effectiveness as a CPD activity and participants’ perceptions of their use of the app with regard to the *effectiveness* outcome.

The CPD-Min app sent out 110 questions to participants throughout the 52 weeks. In total, 40.9% (43/105) of participants responded to more than 90% of the questions, with 15.2% (16/105) completing every question. Participants had an overall high mean completion rate of 75% (SD 33%) throughout the study. However, there was a significant difference in the average response rate before and after the midpoint assessment at 26 weeks (*t*_104_=−5.06; *P*<.001, 2-tailed). On average, the premidpoint response rate was 5 questions higher than the postmidpoint response rate (95% CI −7.07 to −3.05). Normality was assumed by visually inspecting Q-Q plots and considering the larger sample size (n=104) in accordance with the central limit theorem.

### Research Objective 2: Effectiveness of This Tool as a CPD Activity—Effectiveness

#### Overview

The average number of questions answered per participant was 82 (SD 32.9; range 0-110) questions, with a mean correct response of 43 questions per participant (SD 19.5; range 0-76)*.* They spent an average of 35.3 (SD 5.9; range 14-48) seconds to provide an answer to each weekly question. Spearman rank correlation tests (*r_s_*) showed weak positive correlations between questions answered correctly and both participants’ confidence (*r_s_*=0.272; *P*<.001) and relevance (*r_s_*=0.143; *P*<.001) rating of those correct questions.

#### Semistructured Interviews

A total of 15 ANES participants were interviewed from both cohorts. On the basis of the thematic analysis of the interview data, the following themes regarding participants’ thoughts and perceptions on the CPD-Min app were generated: (1) the practical design of the novel educational app facilitated its adoption by clinicians, (2) the app was perceived as a knowledge tool for continuous learning, and (3) the app’s low-stakes testing environment cultivated independent learning attitudes.

#### Theme 1: The Practical Design of the Novel Educational App Facilitates Its Adoption by Clinicians

The convenience and practicality of this novel app facilitated participants’ adoption of the learning tool (second-level of the framework by Moore et al [26] - satisfaction). Participants noted that both the practicality and convenience of having this continuing learning tool in an app format, along with its short time commitment, helped them maintain their participation and engagement throughout the study period and possibly beyond:

There’s a practicality to the app and to the timeframes required by the question-based module, which is interesting and fun. There’s something practical about having bite-size elements of CPD that can be done anywhere at any time, which is very practical.

ID 109

According to participants in the interview, they were consistent with using the app throughout the program, taking approximately 5 minutes to answer questions and review materials if necessary. In addition, most participants found the weekly reminder emails to be useful in prompting them to answer the question right away or to schedule a time to do it during the week. When it came to the design of the app, while most participants noted the ease of use and practicality of it, there were a few suggestions on improving some features within the app. One participant suggested improving access to previous questions and resources by integrating a quick search function to refer to in the future if needed.

### Research Objective 3: Relevance of the Disseminated Information to Physicians’ Practice (Theme 2: The App Was Perceived as a Useful Knowledge Tool for Continuous Learning)

The app provided health care professionals with an opportunity to engage in a continuous learning experience. Most participants mentioned using the app as a tool to identify gaps in their knowledge by reflecting on their scores and the content of the app. The CPD activity was described as a “refresher” of previous knowledge for the licensing exams by several participants. Moreover, a few participants expressed that although some of the subspecialty content was not relevant to their practice, it was useful in identifying subspecialty gaps and providing an overview of clinical topics within different subspecialties in the field:

Some of the content is not necessarily relevant to my practice. But I think as an anesthesiologist, you never know what’s going to come up. So, it’s good to keep a broad overview of things, even if you’re not doing them every day.

ID 85

For some participants, having a broad range of subspecialty topics provided them with a refreshing overview of knowledge regardless of its relevancy to their practice. By contrast, a few participants reported that they would have appreciated a more tailored approach to the content of the app. Specifically, 1 participant reported that the number of questions that did not relate to their practice “did not necessarily increase [their] learning.” As a recommendation, a few participants suggested having the option to choose specific topics of interest at the start of using the app. Moreover, interview participants reported engaging in the supplementary material and feedback provided to them; some used the references provided to them to further explore specific topics they were interested in.

### Research Objective 4: Acceptability Among ANES Residents, Fellows, and Staff of This Novel Tool as an Educational Initiative (Theme 3: The App’s Low-Stakes Testing Environment Cultivates an Independent Learning Attitude)

The app uses low-stakes repeated testing to cultivate lifelong learning among health care professionals. Participants noted that low-stakes and repeated testing design of the app was useful in keeping them engaged throughout the program and further cultivated their lifelong learning:

Doing the CPD is extremely low stakes. I do this because, well, I want to keep lifelong learning. I like to learn. I care to be as good of an Anesthesiologist as I can. And just as well to keep on practicing and learning and reviewing the material. And this, in a way, forces you or at least prompts me to do it, in an easy bite-sized manner. The low stakes is what keeps me in. If I knew that if I failed this, I’d lose my license, I be slightly more worried. This is only win-win. I need to do CPD. I do CPD. It’s easy. Why not?

A109

Similar to the themes generated, findings from the postsurvey confirmed participants’ perceptions during the midpoint interviews, where participants highlighted the effectiveness of the app as an educational tool, as it not only tests their current knowledge but also exposes them to the newest guidelines, studies, and information related to their practice. Concurrently, while exploring the quality of the app’s content in the postsurvey, most respondents (57/75, 76%) were satisfied with the variety of topics offered, and even more participants (64/75, 85%) reported the quality of the questions and supplementary material as above average (ie, excellent or good).

The themes generated from the interview data were further supported by the survey findings (Table 3), as over one-third of the participants (29/75, 39%) reported that they would *always* or *often* check after answering correctly, while even more (57/75, 76%) reported *always* or *often* checking the feedback after incorrect responses. More than half of the participants (43/75, 57%) did not report any more engagement with the app after answering correctly rather than incorrectly. Furthermore, most participants (58/75, 77%) were intrigued to find out the answers to the questions asked of them weekly. Most participants (45/75, 60%) were *occasionally* motivated to seek out additional, related self-directed learning external from the app. The CPD-Min app was reported as a frequent, low-stakes knowledge testing platform by most participants (62/75, 83%). Most participants (65/75, 87%) did not perceive the 2 knowledge assessment weeks to be too stressful or anxiety inducing.

With regard to participants’ learning, on average, participants scored 16% (SD 33.1%) less on their end point knowledge assessment compared to their midpoint knowledge assessment. From the final survey results, most participants (38/41, 93%) reported that the app enhanced their clinical knowledge and helped them identify gaps in their learning (third level of a framework by Moore et al [26] - learning). They also found the knowledge assessments helpful. Furthermore, it provided them with adequate resources to further their learning. For most participating clinicians (35/41, 85%), the app helped them improve or change their practice, and for some (31/41, 76%), it also contributed to their confidence with their clinical knowledge.

**Table 3:** Postsurvey response on user engagement (Likert scale) by users of the CPD By the Minute app (N=75).

	Always or strongly agree, n (%)	Often or agree, n (%)	Occasionally or neither agree nor disagree, n (%)	Rarely or disagree, n (%)	Never or strongly disagree, n (%)
How likely are you to review the supplementary materials provided (eg, key points, references, and critiques) after a correct response?	10 (13)	19 (25)	28 (37)	15 (20)	3 (4)
How likely are you to review the supplementary materials provided (eg, key points, references, and critiques) after an incorrect response?	32 (43)	25 (33)	13 (17)	3 (4)	2 (3)
I was more likely to engage with the app if a previous question was correct rather than incorrect.	2 (3)	4 (5)	26 (35)	19 (25)	24 (32)
The results I received after answering a question motivated additional, related self-directed learning external to the app.	4 (5)	19 (25)	45 (60)	6 (8)	1 (1)
I was intrigued to find out the answers to the questions asked of me.	26 (35)	32 (43)	15 (20)	2 (3)	0 (0)
Participating in either the routine weekly questions or KA^a^ weeks was too stressful and induced anxiety.	0 (0)	2 (3)	8 (11)	26 (35)	39 (52)

^a^KA: knowledge assessment.

### Implementation of the CPD-Min App

With regard to the *implementation* component of the CPD-Min app, the delivery or format of the app was assessed using both the interviews and postsurvey. Participants spoke positively about the CPD-Min app’s format, content, and delivery method (Table 4). Participants noted that the weekly question count was manageable within their workload and helped them stay consistent in using the app. Several participants also highlighted that the weekly reminders were beneficial in prompting them to complete their questions for the week. Moreover, the questions were mostly relevant to realistic case scenarios that were applicable to their practice. Finally, many participants positively described the appropriateness of the format for CPD and continuous learning.

Likewise, the findings from the postsurvey regarding the quality and usability of the app concur with the abovementioned findings from the midpoint interviews. Most postsurvey participants (49/75, 65%) reported the format and layout (eg, character count, image, and font size) of the questions in the app to be appropriate for a time-sensitive response. The app’s usability was measured using the SUS, and the mean scale score as reported by participants was 77.7 (SD 6.7; range 60-88). The usability score was rated on a scale of 100, with an average score being 68 and a score >80 considered above average [24]. A score of 77 indicated that the CPD-Min app was fairly usable; however, there were still areas that could be improved to enhance the overall user experience.

While majority of participants (51/75, 68%) found the peer reflection feature of the app (ie, being able to compare their performances to their peers) helpful and about half of the participants (30/75, 40%) thought it was important, most participants (54/75, 72%) reported *never* discussing the results or material with their peers. Almost one-third of the participants (21/75, 28%) reported discussing their results with their peers from once a week to less than once a month. This was concurrent with findings from the interview, as most interview participants reported never discussing the material or content from the app with colleagues. When asked to elaborate, a few interview participants highlighted that the CPD activity in this app format did not facilitate discourse and discussion among peers and colleagues. Participants found themselves using the app as a self-guided learning tool as opposed to other CPD activities, such as conferences. A few participants (2/15, 13%) suggested integrating an in-app feature that allowed interaction or discussion with other CPD-Min users.

**Table 4:** Postsurvey responses on quality assessment (Likert scale) by users of the CPD By the Minute app (N=75).

	Strongly agree or excellent, n (%)	Agree or good, n (%)	Neither agree nor disagree or average, n (%)	Disagree or below average, n (%)	Strongly disagree or poor, n (%)
I was satisfied with the variety of topics and subject matter.	12 (16)	45 (60)	12 (16)	4 (5)	2 (3)
The quality of questions and supplementary materials offered was (poor to excellent).	21 (28)	43 (57)	9 (12)	2 (3)	0 (0)
While answering a given question, I felt the format and layout were appropriate for a time-sensitive response.	9 (12)	40 (52)	8 (11)	14 (19)	4 (5)

### Maintenance

CPD-Min program’s *maintenance* feature focused on the individual level, exploring participants’ likelihood of continued use of the CPD-Min app and recommendation to peers and colleagues (Table 5). Most participants (63/75, 84%) who reported that they were likely to continue using the CPD-Min app, also reported that they would continue using it as an ongoing CPD activity (61/75, 81%). Most participants (67/75, 89%) reported that they would likely continue using the app if they received section 3 MOC (self-assessment) CME credits. Similarly, 89% (67/75) of the participants reported that they would continue using the CPD-Min app for >12 months. Most participants (52/75, 69%) found the app to be an effective learning tool for their practice, with about 28% (21/75) reporting that the app was *somewhat* effective as a learning tool. In terms of recommending the app to colleagues, 79% (59/75) of the participants said that they would recommend the app to their colleagues, and 81% (61/75) said that they would specifically recommend it to residents and fellows.

**Table 5:** Postsurvey responses on appeal and effectiveness (Likert scale) by users of the CPD By the Minute (CPD-Min) app (N=75).

	Very likely or effective, n (%)	Likely or effective, n (%)	Somewhat, n (%)	Not very, n (%)	Not at all, n (%)
How likely are you to continue using the CPD-Min app?	43 (57)	20 (27)	8 (11)	4 (5)	0 (0)
How likely are you to continue using the app as an ongoing CPD^a^ activity?	40 (53)	21 (28)	9 (12)	4 (5)	0 (0)
Would you likely continue using the app if you continued receiving section 3 MOC^b^ (self-assessment) CME^c^ credits?	53 (71)	14 (19)	6 (8)	1 (1)	0 (0)
How effective is this as a learning tool for your practice?	19 (25)	33 (44)	21 (28)	2 (3)	0 (0)
How likely are you to recommend the app to your colleagues?	34 (45)	25 (33)	15 (20)	1 (1)	0 (0)
How likely are you to recommend the app to residents or fellows?	36 (48)	25 (33)	9 (12)	3 (4)	2 (3)

^a^CPD: continuing professional development.

^b^MOC: Maintenance of Certification.

^c^CME: continuing medical education.

## Discussion

### Principal Findings

The CPD-Min app disseminated 110 peer-reviewed questions to 105 ANES clinicians over the span of 52 weeks. Upon the evaluation of this app as a CPD tool, three major themes were identified: (1) the practical design of the educational app facilitated its adoption by clinicians, (2) the app was perceived as a useful knowledge tool for continuous learning, and (3) the app’s low-stakes testing environment cultivated independent learning attitudes. In addition to clinicians’ positive perceptions of the CPD-Min app, they noted that using it helped them identify gaps in their clinical knowledge. The implementation of a longitudinal self-assessment activity such as CPD-Min has various strengths, enabling health care professionals to assess their own knowledge and identify areas where they may need to improve. CPD is essential to maintaining and improving clinical practice among physicians. It allows health care professionals to stay up-to-date with the latest developments in their field and to acquire new skills and knowledge that can help them deliver better care to their patients. However, traditional CPD activities are associated with several limitations in their implementation (eg, lack of time, cost, and reliance on self-assessments) and rate of dissemination [2-4].

Among the ANES clinicians who participated in the survey, 63% (26/41) were aged between 35 and 55 years and 80% (33/41) were male. The age of this sample was comparable to the general ANES population according to reports from the Canadian Medical Association, noting that >50% of the Canadian ANES population was aged between 35 and 55 years [27]. However, our sample had slightly more ANES clinicians identifying as a cisgender man (33/41, 80%) compared to the general population (67%) [27]. Similarly, our population’s work setting was comparable to the general Canadian ANES population. According to the 2019 Canadian Medical Association report, most ANES clinicians reported academic health science centers and community hospitals as their top primary work settings [27].

The CPD-Min pilot program had high engagement as participating ANES clinicians had an average of 75% (SD 33%) completion rate through the entire year of the study program. The average clinician participation rate in the CPD-Min program was consistent with participation levels from 10 previously evaluated educational delivery programs for health care professionals, as explored in a systematic review by Phillips et al [28]. Clinician participation levels in self-assessment CPD programs could be attributed to a number of factors, including clinical relevance, optimal number of questions per day, and their spacing [29]. In addition, the decrease in participant response rate over the course of the program is not an uncommon occurrence, as the drop in participation over time could be due to fatigue, lack of motivation, or other personal or external factors [30-32].

With the CPD-Min app, clinicians’ adoption of the app and participation in the program could be attributed to the ease of use and ability to quickly adapt to this microlearning pedagogy method. The app provided specialized physicians with an opportunity to participate in continuous learning in a flexible and accessible manner that encouraged sustained engagement [33]. The microlearning style presented by this CPD app was a helpful format for continuous learning and development for physicians, as they were able to be consistent with using the app throughout the program, taking approximately 5 minutes to answer questions and review materials if necessary [34]. Clinicians’ responses to the CPD-Min app echoed key takeaways of mobile-microlearning pedagogy within workplaces that highlight the usefulness and demand of just-in-time learning, which provides clinicians with bite-sized lessons that can be immediately applied within their clinical practices [33,35-37]. In addition, this nontraditional mode of CPD allows for spatial and temporal flexibility among clinicians with demanding workloads who are seeking to engage in CPD. For instance, participating clinicians report being able to engage in learning “on the go,” allowing them to seamlessly integrate their continuous learning within their workflows. Participating clinicians noted that they were always able to find time to do it throughout their working days (eg, during a coffee break or free time waiting in the operating room).

The app’s low-stakes testing environment encouraged independent learning attitudes. Participants reported low anxiety and stress while using the app and actively engaged with supplementary resources and feedback provided after each question. While the use of the app seemed to encourage continued learning through the just-in-time low-stakes learning environment, the structure of the app did not facilitate discussion of the material with colleagues, as highlighted by participants. The facilitation of discussion and interaction during CPD uptake provides clinicians the opportunity to reflect on their performance and knowledge in reference to their peers. Although the CPD-Min app did not facilitate collaboration and peer interaction, a key feature of the app was its weekly summary. This summary, detailing participants’ and their colleagues’ performance, served as a tool for reflection and played a crucial role in motivating clinicians to remain actively engaged with the app. This finding aligns with previous literature that emphasizes the importance of clinician reflection in CPD activities, helping them identify gaps in performance and knowledge [38,39].

The CPD-Min app disseminated information to clinicians in a timely manner [40]. The app used the declarative knowledge state with key features such as feedback, key critiques, and references to translate knowledge to clinicians’ practice [41]. This finding is consistent with a recent study that reported the benefits of immediate feedback for knowledge retention [42]. With the rapidly evolving research knowledge in health care, there seems to be a slow rate in knowledge uptake and dissemination into practice [43]. The well-received engagement with the CPD-Min app and uptake in knowledge, as well as engagement with the resources, indicates the potential of using such a mobile question-based app to translate and disseminate new and innovative knowledge in the field. Repeated testing has been shown to enhance knowledge retention and promote higher-level cognitive processing more effectively than traditional learning methods [42,44].

The CPD-Min app aimed to address gaps in CPD self-assessment for clinicians and facilitate their ability to identify knowledge gaps using just-in-time learning, which prioritizes meeting clinicians’ current learning needs. While the purpose of this app was not to test knowledge, it served the purpose of a self-assessment tool for clinicians. Many participants (38/41, 93%) noted that the app helped them identify knowledge gaps and enhanced their clinical knowledge. In addition to participants’ self-assessment of their knowledge, they reported that the app played a role in improving their practice by guiding their learning and highlighting the overall effectiveness of the intervention. Similar findings were reported in a pilot study of family physicians, indicating that most clinicians continued to integrate the longitudinal assessment as a form of continuous learning and changed their practice because of participating in the program [45]. Interestingly, another study found that in high-stakes assessment, participants were more accurate but less confident in their responses [44]. Low confidence could be attributed to the high-stakes nature of the assessment [44]. Overall, the results of the CPD assessment program suggested that the CPD-Min app was an important learning pathway for anesthesia clinicians and was perceived as a useful knowledge tool for continuous learning. However, further research is needed to explore the effectiveness of the app in promoting knowledge retention and the accuracy of physicians’ self-assessment of their knowledge. Integrating this form of longitudinal assessment into organizational workflows could enhance physician performance and, ultimately, improve patient outcomes.

Participants found the CPD-Min app to be an effective tool for their practice as it helped them identify knowledge gaps in their practice. Overall, based on the findings from both the survey and interviews, participants perceived the app to be useful for their practice. Regarding the perceived ease of use, the high SUS score suggests that the app is well designed and user-friendly, which could lead to its adoption and integration into clinical practice.

### Limitations

This study has a few limitations, which should be acknowledged. The generalizability of the study is limited because the study population focused only on one medical discipline (ie, ANES), and thus, the findings may not be applicable to other medical specialties. Future studies should replicate the study design in various medical fields to determine the app’s generalizability and usefulness in different contexts. In addition, study recruitment and intervention were administered during the COVID-19 pandemic, which limited the recruitment diversity of clinician roles and significantly impacted the participants’ engagement with the study’s intervention. Although the intervention was conducted remotely, the COVID-19 pandemic may have influenced clinicians’ work schedule and stress levels, ultimately affecting their overall engagement with the study intervention. Another limitation of the study was that the question development process was labor intensive. Large language models could be used to rapidly prototype the questions in the future. Finally, due to the randomized nature of the questions disseminated through the app, inferences could not be made from the knowledge assessment findings, as it would require controls such as exact questions and spacing in administration. The highlighted limitations should be considered when interpreting these findings. Future research should aim to address these limitations to provide a more comprehensive understanding of the app’s effectiveness as a continuous learning tool for clinicians.

### Conclusions

The CPD-Min app was positively received and accepted as an educational initiative by ANES clinicians, and its practical design encouraged continued use of the app by the clinicians. Furthermore, the information was extremely relevant to participants’ practices while also allowing them to brush up on knowledge from subspecialties they might not frequently practice. The CPD tool allowed ANES clinicians to recognize knowledge gaps and promote continuous learning. This app uses a practical longitudinal approach to incorporating CPD into the workflow and provides opportunities to facilitate the dissemination of new clinical information and updates across health care organizations. Integrating mobile microlearning activities into the workflow allows health care professionals to engage in ongoing learning and development more easily, leading to improved performance and outcomes. While the initial results are promising, further evaluation of the app is necessary to fully understand its long-term implications and the sustainability of the app over time. Another key area of evaluation is assessing practice changes among clinicians using the app and its impact on organizational workflows. In summary, while the CPD-Min app has shown promise in enhancing learning and knowledge retention, further evaluation is needed to understand its long-term implications. This includes examining the sustainability of the CPD activity and assessing the impact on clinical practice and patient outcomes.

## Appendix

Multimedia Appendix 1 Postsurvey questions.

## Abbreviations

ANES: American Board of Anesthesiology
CME: continuing medical education
CPD: continuing professional development
CPD-Min: CPD By the Minute
MCQ: multiple-choice question
MOC: Maintenance of Certification
RE-AIM: Reach, Effectiveness, Adoption, Implementation, and Maintenance
REDCap: Research Electronic Data Capture
RCPSC: Royal College of Physicians and Surgeons
SUS: System Usability Scale
TEL: test-enhanced learning
UofT: University of Toronto
UHN: University Health Network
