# Supplementary material for: Innovative Mobile App (CPD By the Minute) for Continuing Professional Development in Medicine: Multimethods Study
Source: JMIR Med Educ. 2025 Jul 23;11:e69443. doi: 10.2196/69443 (PMC12329386; doi:10.2196/69443)
Supplement: Multimedia Appendix 1 [file mededu_v11i1e69443_app1.docx]

**Multimedia Appendix: Post Survey Questions**

**Section 1: Study Identification Code**

Please **select one response** for each question unless otherwise stated.

1. Please enter your unique study identification code provided to you by a member of the research team. To retrieve this information, please send an email to CPD by the Minute research staff.
2. __________________

**Section 2: CPD By the Minute Application Effectiveness & Appeal**

Please **select one response** for each question.

1. How likely are you to continue using CPD By the Minute?

- Very likely
- Likely
- Somewhat
- Not very
- Not at all

2. How likely are you to continuing using this as an ongoing continuing professional development activity?

- Very likely
- Likely
- Somewhat
- Not very
- Not at all

3. Would you be more likely to continue using CPD By the Minute if you continued to receive section 3 MOU (self-assessment) CME credits?

- Very likely
- Likely
- Somewhat
- Not very
- Not at all

4. For how long do you believe you would continue to use CPD By the Minute?

- Less than 1 month
- 1 – 3 months
- 4 – 7 months
- 8 – 12 months
- Over 12 months

5. How effective is this as a learning tool for your practice?

- Very effective
- Effective
- Somewhat
- Not very effective
- Not at all effective

6. How likely are you to recommend CPD By the Minute to your colleagues?

- Very likely
- Likely
- Somewhat
- Not very
- Not at all

7. How likely are you to recommend CPD By the Minute to residents or fellows?

- Very likely
- Likely
- Somewhat
- Not very
- Not at all
- Not applicable

9. How much would you be willing to pay for CPD By the Minute annually?

- Less than $49.99
- $50.00 - $99.99
- $100.00 - $149.99
- Greater than $150.00
- I would only use CPD By the Minute if it were free.
- Please expand on your choice below:
- _______________________________________________________

**Section 3: Influence of Peers/ Colleagues**

1. It was helpful to know how well I was performing compared to my peers/ colleagues:
2. Strongly disagree
3. Disagree
4. Neither disagree/ agree
5. Agree
6. Strongly agree
7. It was important to perform as well or better compared to my peers/ colleagues:
8. Strongly disagree
9. Disagree
10. Neither disagree/ agree
11. Agree
12. Strongly agree
13. I talked to a peer(s)/ colleague(s) about my results during the study:
14. More than once a week
15. Once a week
16. Once a month
17. Less than once a month
18. Never

**Section 4: User Engagement Behavior**  

1. How likely were you to review the supplementary materials provided (e.g. key points, references, critiques) after a correct response?
2. Always
3. Often
4. Occasionally
5. Rarely
6. Never
7. How likely were you to review the supplementary materials provided (e.g. key points, references, critiques) after an incorrect response?
8. Always
9. Often
10. Occasionally
11. Rarely
12. Never
13. I was more likely to engage with the app (by answering the next/ subsequent question) if a previous question was correct rather than an incorrect response:
14. Strongly disagree
15. Disagree
16. Neither disagree/ agree
17. Agree
18. Strongly agree
19. The results I received after answering a question motivated additional, related self-directed learning external to the application:
20. Always
21. Often
22. Occasionally
23. Rarely
24. Never
25. I was intrigued to find out answers to the questions asked of me:
26. Always
27. Often
28. Occasionally
29. Rarely
30. Never
31. Participating in either the routine weekly questions or ‘Knowledge Assessment’ weeks was too stressful, induced anxiety:
32. Always
33. Often
34. Occasionally
35. Rarely
36. Never
37. CPD By the Minute is a *frequent, low-stakes* knowledge testing platform.
38. Strongly disagree
39. Disagree
40. Neither disagree/ agree
41. Agree
42. Strongly agree

**Section 4: Quality Assessment**  

1. I was satisfied with the variety of topics/ subject matter offered:
2. Strongly disagree
3. Disagree
4. Neither disagree/ agree
5. Agree
6. Strongly agree
7. The quality of questions and supplementary materials offered were:
8. Excellent
9. Good
10. Average
11. Below average
12. Poor
13. While answering a given question, I felt the format/ layout was appropriate for a time sensitive response (e.g. character count, image and font size, etc.):
14. Strongly disagree
15. Disagree
16. Neither disagree/ agree
17. Agree
18. Strongly agree

**Section 4: App Design Considerations**

1. Preferred method to communicated/ notify you of new questions. Select all that apply:
2. Email
3. Text messaging/ SMS
4. Calendar reminders
5. Automated phone call
6. No reminders or notifications
7. Would you prefer to have had the option to personalize when and how frequent the reminders to answer questions were administered?
8. Yes
9. No
10. Did the personal mobile device you used limit your ability to take part in the study:
11. Yes
12. No

**Section 4: System Usability Scale (SUS)**

Please indicate the extent of your agreement with each of the following statements by circling the appropriate number. Please record your immediate response to each statement.

|  | Strongly disagree |  |  |  | Strongly agree |
| --- | --- | --- | --- | --- | --- |
| 1. I think that I would like to use CPD By the Minute frequently | 1 | 2 | 3 | 4 | 5 |
| 2. I found CPD By the Minute unnecessarily complex | 1 | 2 | 3 | 4 | 5 |
| 3. I thought CPD By the Minute was easy to use | 1 | 2 | 3 | 4 | 5 |
| 4. I think that I would need the support of a technical person to be able to use CPD By the Minute | 1 | 2 | 3 | 4 | 5 |
| 5. I found the various functions in CPD By the Minute were well integrated | 1 | 2 | 3 | 4 | 5 |
| 6. I thought there was too much inconsistency in CPD By the Minute | 1 | 2 | 3 | 4 | 5 |
| 7. I would imagine that most people would learn to use CPD By the Minute very quickly | 1 | 2 | 3 | 4 | 5 |
| 8. I found CPD By the Minute very cumbersome to use | 1 | 2 | 3 | 4 | 5 |
| 9. I felt very confident using the CPD By the Minute | 1 | 2 | 3 | 4 | 5 |
| 10. I needed to learn a lot of things before I could get going with CPD By the Minute | 1 | 2 | 3 | 4 | 5 |

**Section 3: Orientation to Lifelong Learning**

Please consider the definition below before responding to the next set of questions.

1. Modified Jefferson Scale of Health Professionals Lifelong Learning

Please indicate the extent of your agreement with each of the following statements by circling the appropriate number.

|  | **Strongly Disagree** | **Disagree** | **Agree** | **Strongly Agree** |
| --- | --- | --- | --- | --- |
| a) Searching for the answer to a question is, in and by itself, rewarding | 1 | 2 | 3 | 4 |
| b) Lifelong learning is a professional responsibility of all physicians | 1 | 2 | 3 | 4 |
| c) I enjoy reading articles in which issues of my professional interest are discussed | 1 | 2 | 3 | 4 |
| d) I routinely attend annual meetings of professional medical organizations | 1 | 2 | 3 | 4 |
| e) I read professional journals at least once every week | 1 | 2 | 3 | 4 |
| f) I routinely search computer databases to find out about new developments in my specialty | 1 | 2 | 3 | 4 |
| g) I believe that I would fall behind if I stopped learning about new developments in my profession | 1 | 2 | 3 | 4 |
| h) One of the important goals of health professions’ education is to develop students’ lifelong learning skills | 1 | 2 | 3 | 4 |
| i) Rapid changes in medical science require constant updating of knowledge and development of new professional skills | 1 | 2 | 3 | 4 |
| j) I always make time for self-directed learning, even when I have a busy work schedule and other professional and family obligations | 1 | 2 | 3 | 4 |
| k) I recognize my need to constantly acquire new professional knowledge | 1 | 2 | 3 | 4 |
| l) I routinely attend optional continuing medical education programs to improve patient care | 1 | 2 | 3 | 4 |
| m) I take every opportunity to gain new knowledge/skills that are important to my profession | 1 | 2 | 3 | 4 |
| n) My preferred approach in finding an answer to a question is to search the appropriate computer databases | 1 | 2 | 3 | 4 |

You have now completed the survey.  
 
Thank you for your time and input.
